# Variations in different preceding crops on the soil environment, bacterial community richness and diversity of tobacco-planting soil

**DOI:** 10.3389/fmicb.2024.1389751

**Published:** 2024-05-28

**Authors:** Ming Liu, Rujun Xue, Dexun Wang, Yanxia Hu, Kaiyuan Gu, Liu Yang, Jie Zhao, Shuyue Guan, Jiaen Su, Yonglei Jiang

**Affiliations:** ^1^College of Agronomy and Biotechnology, Southwest University/Engineering Research Center of South Upland Agriculture, Ministry of Education, Chongqing, China; ^2^Dali Prefecture Branch of Yunnan Tobacco Company, Dali, Yunnan, China; ^3^Weishan City Branch of Yunnan Tobacco Company, Weishan, Yunnan, China; ^4^Yunnan Academy of Tobacco Agricultural Sciences, Kunming, China

**Keywords:** preceding crops, tobacco-planting soil, nutrient status, bacterial community, bacterial diversity

## Abstract

Tobacco (*Nicotiana tabacum* L.) is a major cash crop, and soil quality played a significant role in the yield and quality of tobacco. Most farmers cultivate tobacco in rotation with other crops to improve the soil characteristics. However, the effects of different previous crops on the soil’s nutrient status and bacterial community for tobacco cultivation still need to be determined. Three treatments were assessed in this study, i.e., tobacco-planting soil without treatment (CK), soil with barley previously cultivated (T_1_), and soil with rapeseed previously cultivated (T_2_). The soil physical and chemical properties and the 16S rRNA gene sequence diversity of the bacterial community were analyzed. The effects of different crops on the physical and chemical properties of tobacco-planting soil and the diversity and richness of the bacterial community were comprehensively discussed. The results of this study showed that different previously cultivated crops altered the nutrient status of the soil, with changes in the ratio of NH_4_^+^-N to NO_3_^−^-N having the most significant impact on tobacco. In CK, the ratio of NH_4_^+^-N to NO_3_^−^-N was 1:24.2, T_1_–1:9.59, and T_2_–1:11.10. The composition of the bacterial community in tobacco-planting soil varied significantly depending on the previously cultivated crops. The richness and diversity of the bacterial community with different crops were considerably higher than without prior cultivation of different crops. The dominant bacteria in different treatments were *Actinobacteriota, Proteobacteria, and Chloroflexi* with their relative abundance differed. In conclusion, our study revealed significant differences in nutrient status, bacterial community diversity, and the richness of tobacco-planting soil after the preceding cultivation of different crops. Suitable crops should be selected to be previously cultivated in tobacco crop rotations in near future for sustainable agriculture.

## Introduction

Tobacco (*Nicotiana tabacum* L.) is a significant cash crop in Yunnan Province, China, and the productivity, chemical properties and their proportions decide tobacco leaves quality. Because of the mountainous area and limited agricultural land, continuous tobacco cultivation is widespread in Yunnan Province. Continuous monoculture results in soil nutrient imbalances, deterioration of physical and chemical properties, aggravation of soil-borne diseases, and disturbance of soil ecological balance (Tang et al., 2020). The long-term continuous cropping of tobacco led to severe disease occurrence and yield reduction (Chen et al., 2023). Soil microorganisms are a vital part of the soil ecosystem. They participate in entire life processes, such as organic matter decomposition, nutrient cycling, and energy transfer in soil, and play a key role in soil remediation and soil ecological stability (Verma et al., 2020, 2023, 2024; Hemkemeyer et al., 2021; Adomako et al., 2022). Long-term continuous cropping changes the soil ecological environment, resulting in corresponding changes in the soil microbial community (Wu et al., 2022; Paes da Costa et al., 2024). The number of soil bacteria increased yearly after continuous cropping for 2 to 10 years during isolation and cultured soil microorganisms of different planting patterns (Jaiswal et al., 2022). The number of bacteria significantly downregulated after more than 10 years of continuous cropping (Verma et al., 2023, 2024). The number of azotobacter and actinomycetes enhanced with continuous cropping yearly. The soil microbial diversity index and uniformity decreased under continuous cropping systems. Tan et al. (2021) also found that under different durations of continuous cropping, significant differences in the abundance of bacteria and fungi in tobacco fields but not in the abundance of actinomyces. The abundance of fungi in the soil increased, bacteria decreased, and actinomyces increased slightly but not significantly during continuous cropping system. Continuous cropping affected soil microbial diversity, increased soil pathogens, led to flue-cured tobacco soil diseases, and adversely affected the health of soil cultivated with tobacco. Long-term continuous cropping of tobacco caused changes in the soil microbial community, a transition of soil microbiota from bacterial to fungal dominance and soil fertility deterioration (Chen et al., 2023; Paes da Costa et al., 2024).

Nowadays, several studies have shown that the rotation of flue-cured tobacco with other crops can effectively reduce the consequences of continuous cropping, improve the quality of flue-cured tobacco, reduce the occurrence of diseases, and improve the quality of the soil (Fortnum et al., 2001; Jiang et al., 2022). Soil pH, organic matter, total potassium, available boron, zinc, CEC, and exchangeable magnesium under crop rotation were increased compared to soil under continuous cropping (Verma et al., 2021a, 2022a,b; Chen and Zeng, 2022). Previous studies on crop rotation and intercropping of tobacco and rice, corn, garlic, green manure, etc., found that crop rotation can effectively reduce the impact of tobacco continuous cropping (Huang et al., 2019, 2022; Wang et al., 2022, 2023). Especially for soil microorganisms, crop rotation can effectively improve the population properties of soil microorganisms, as imbalances of soil microbial community are a common phenomenon caused by continuous cropping. Soil dominated by the rhizospheric bacteria as a biological index of good soil quality (Chen et al., 2023). The crop rotation can alter the status and composition of bacterial and fungal communities in soil, protect the excessive proliferation of some microbial populations, such as pathogens and harmful microorganisms that are prone to occur during continuous cropping, and promote the growth of beneficial microorganisms. Different plant species attract and nourish different microbial communities in their rhizosphere, and crop rotation can contribute to balance the soil microbial diversity and ecological balance (Xi et al., 2021; Verma et al., 2021b, 2022c; Woo et al., 2022; Wang et al., 2023; Paes da Costa et al., 2024).

Crop rotation affects the functions of soil microorganisms, including their roles in nutrient cycling, decomposition of organic matter, disease resistance capacity, and plant growth promotion. Different plant root exudates and residues provide different carbon and energy sources, affecting microorganisms metabolic activities, functional properties and efficiency. By changing the microbial activity and nutrient cycling process, the fixation and loss of certain nutrients can be reduced, and improved the availability of nutrients. Some of the crops can enhance the biological availability of nutrients such as nitrogen, phosphorus, and potassium in the soil through their root exudates or residues (Chen et al., 2023; Paes da Costa et al., 2024).

Previous studies have demonstrated that selecting previously cultivated crops affects soil microorganisms and nutrient status differently. In order to better assess the effects of different precursor crops on the tobacco-planting soil environment, richness and diversity of the bacterial community. The nutrient status and the bacterial 16S rRNA gene sequence diversity in tobacco-planting soil previously cultivated with different crops and analyzed the changes in soil environmental factors and bacterial species composition jointly. We hypothesized that different previously cultivated crops would differentially affect the diversity and richness of bacterial communities in tobacco-planting soil and dominant bacterial species would be altered due to variation in the soil properties.

## Materials and methods

### Experiment design

This experiment was conducted in December 2022 in Weishan County, Dali Prefecture (E 100°30′, N 25°23′, altitude 2000 m), Yunnan Province, China. In 2023, Weishan country had an average annual precipitation of 823.3 mm, temperature 17°C, and photoperiod of 2,346 h. Physical and chemical properties of the rhizospheric soil were as soil bulk density (1.21 g cm^−3^), pH (6.47), organic matter content (28 g kg^−1^), total nitrogen (1.68 g kg^−1^), total phosphorus (1.46 g kg^−1^), total potassium (34.54 g kg^−1^), available phosphorus (18.13 mg kg^−1^), available potassium (270.23 mg kg^−1^), and alkaline hydrolyzable nitrogen content (35.23 mg kg^−1^), respectively.

The field demonstrations was conducted in a randomized block design (RBD) with three treatments, each with three replicates (*n* = 3). Each replicate plot covered an area of 100 m^2^ (10*10 m). All preceding crops in the plots were tobacco cultivar Hongda. Different treatments were applied as CK- no previous cultivation of any crop, T_1_- previous cultivation of barley (cv. Kunlun 15) and T_2_- previous cultivation of rapeseed (cv. Huayou 5). Cultivars of Kunlun 15 and Huayou are widely used as local varieties. Barley and rapeseed were sown in December 2022. Rapeseed was planted with a row spacing of 25 cm and plant spacing of 20 cm and barley planted with a row spacing of 25 cm and plant spacing of 10 cm. Basal fertilizers were applied before planting as per crop recommendation. Total nitrogen fertilizer was applied (187.5 kg/hm^2^) for rapeseed and barley plants, splited into two different doses, such as 80% as basal and 20% applied during flowering stage. Compound fertilizer (N:P: K = 15:15:15) was applied at 62.5 kg/hm^2^ as a basal application dose. The CK had no previous crop cultivation and no fertilizer application. Tobacco fertilizer was provided by the Dali Prefecture Tobacco Company of Yunnan Province, China for better tobacco crop cultivation, and its application rate was 750 kg/hm^2^ and other management strategies followed by local standards. Crop harvested in the month of May 2023.

### Determination of soil properties

Soil samples were collected after crop harvest. For each treatment, ten residual crop remnants were selected after harvesting. Their roots were excavated, and remove soil particle on the root surfaces. A gentle brushing was used to remove and collect the rhizosphere soil still adhering to the roots. These samples (*n* = 3) were frozen in liquid nitrogen to determine the soil diversity and richness of bacterial communities. The other samples (*n* = 3) were placed in a cool, shaded area for air drying to determine the physio-chemical properties of soil.

Soil NH_4_^+^-N and NO_3_^−^-N were determined in a 0.01 mol·l^−1^ CaCl_2_ solution by an automatic flow analyzer (AA3, SEAL, Germanly). The total nitrogen was determined with H_2_SO_4_ and catalysts (CuSO_4_ and tin powder) and then on a flow analyzer (AA3, SEAL, Germanly). The total phosphorus and potassium of the soil were determined after treatment with H_2_SO_4_ and HCLO_4_, observed at 700 nm wavelength on an automatic enzyme label (Infinite 2000, Tecan, Switzerland), and potassium by flame photometer. Air-dried (5 g) soil was weighed and placed into a triangular bottle, 50 mL ammonium acetate was added, and the solution was placed in a shaker (30 min), then filtered for observation. A flame photometer determined the content of potassium in the soil sample. Soil pH was determined by a pH-4 portable pH meter (soil-to-water ratio 2.5:1) according to Liu et al. (2022).

Rhizospheric soil samples were collected from tobacco-growing soil previously planted with three different preceding crops. Following the manufacturer’s instructions, soil bacterial DNA was extracted from 0.5 g of soil using the PowerSoil^®^ DNA Isolation Kit (Mobio, AL, United States). DNA quantity and quality were assessed by NanoDrop 2000 (Thermo Fisher Scientific, Wilmington, DE, United States) and Qubit 3.0 spectrophotometers. The integrity of the extracted DNA was evaluated on 2% agarose gel electrophoresis (Fatima et al., 2014). The 341F (5′-ACT CCT ACG GGA GGC AGC AG-3′)/806R(5′-GGA CTA CHV GGG TWT CTA AT-3′) and ITS1-1 (5′-CTT GGT CAT TTA GAG GAA GTA A-3′)/ITS1-2 (5′-GCT GCG TTC TTC ATC GAT GC-3′) primer pairs were used to amplify the V3-V4 regions of the bacterial 16S rRNA gene. The PCR reaction was follows as 4 μL 5 × FastPfuBuffer, 2 μL dNTP (2 mmoL/L), 5 μmoL/L forward and reverse primers at 0.8 μL each, 2 μL DNA template, 0.4 μL 5 U/μl Taq polymerase, and ddH_2_O added (20 μL). The reaction conditions were: initial denaturation at 95°C for 3 min, and then denaturation at 95°C for 30 s, annealing at 55°C for 30 s, extension at 72°C for 30 s, repeated for 30 cycles, followed by extension at 70°C for 5 min, and preservation at 4°C. PCR amplification products from each sample replicate were mixed and purified using an AxyPrep DNA (Axygen Biosciences, Union City, CA, USA) gel recovery kit. Quantifluor TM-ST (Promega Corporation, Madison, WI, USA) blue fluorescence quantification system was used for quantitative detection (Morita and Akao, 2021). The final sequencing library was sent to Shanghai Meiji Biotechnology Co., Ltd. for Illumina MiSeq sequencing analysis. Sequence data were deposited in the NCBI SRA database (Accession no. SUB14128373).

### Data analysis

Raw reads with ambiguous bases, an average Phred score of less than 20, and a length of less than 10 bp were filtered out using Trimmomatic software (v 0.36). The chimeric sequences were also identified and removed using the UCHIME software (v 4.2.40). The bacterial operational taxonomic units (OTUs) were clustered at 97% sequence similarity using UPARSE (v 7.0.1090). Subsequently, the bacterial OTUs were annotated using RDP Classifier v 2.2 (Ribosomal Database Project) against the Greengenes (v 201,304) database and the UNITE (v 7.2) database. Venn plot was used to demonstrate the number of unique and common OTUs in different groups using the ‘VennDiagram’ package (R v3.1.1). The alpha diversity of the bacterial communities was assessed by the Chao 1 (species richness) and Shannon (species diversity) indices to analyze the genetic diversity of each group using MOTHUR (v 1.31.2). Moreover, principal coordinate analyses (PCoA) were performed in QIIME software (v 1.80) to reflect the beta diversity of the microbial community. Assess the similarity in the community among the different groups along with the Bray–Curtis distance matrix (Caporaso et al., 2010). Furthermore, linear discriminant analysis (LDA) effect size (LEfSe) was applied to detect different taxa (LDA scores greater than 2.0 at a *p* < 0.01) with significantly differential abundance in the Galaxy online analytics platform.

Statistical analysis of the alpha diversity indices of the bacteria was performed with Tukey’s Honest Significant Difference (HSD) test by R package (v 3.5.3) (*p* < 0.05). The correlation analysis of alpha diversity indices between bacteria was performed using the function ‘cor. One-way analysis of variance (ANOVA) was performed to analyze the impact of different crops on the rhizospheric microbial composition using SPSS. Mantel tests were performed to assess the correlation between rhizosphere microbial communities and soil physical and chemical properties, as well as temperature using the ‘vegan’ package (*p* < 0.05), respectively. In addition, PERMANOVAs were used to assess the effects of the different crops on bacterial communities based on the Bray–Curtis distance using the ‘vegan’ package (*p* < 0.05). The data were analyzed using the online tools of the Majorbio Cloud Platform.^1^

## Results

### Nutrient status of the tobacco-cultivated soil

**Figure 1:**
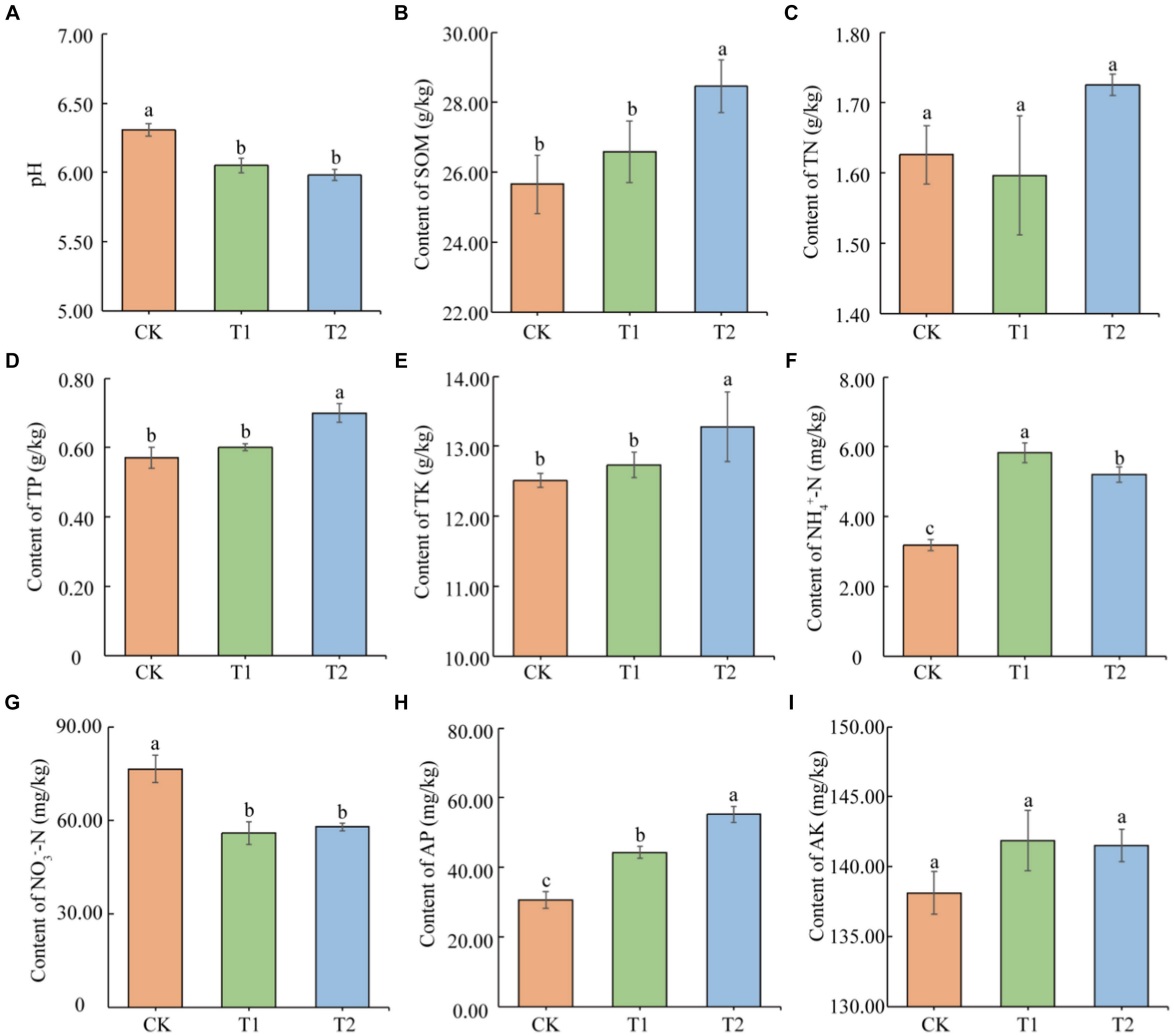
Physical and chemical properties of tobacco-planting soil previously cultivated with different crops. **(A)** soil pH; **(B)** soil SOM; **(C)** soil total nitrogen; **(D)** soil total phosphorus; **(E)** soil total potassium; **(F)** soil NH_4_^+^-N; **(G)** soil NO_3_^−^-N; **(H)** soil available phosphorus; **(I)** soil available potassium. Different letters indicate significant differences between treatments (*p* < 0.05).

Based on the present result findings, the nutrient status of tobacco-growing soil was significantly affected by the different previously cultivated crops (Figure 1). Without planting any crops could effectively slow down the degree of soil acidification. Although the soil pH under the different treatments was acidic. The CK treatment was found higher (4.16 and 5.42%) soil pH than other treatments. At the same time, increased the organic matter content in the T_2_ tobacco planting soil (10.91 and 7.05%) higher than CK and T_1_, respectively. Meanwhile, T_2_ enhanced the TP content in the tobacco-planting soil (22.80 and 16.67%) as compared to CK and T_1_, respectively. T_2_ also significantly increased the soil TK content, which was 6.12 and 4.23% higher than CK and T_1_ treated plants, respectively. There were significant differences in the content of NH_4_^+^-N in the tobacco-planting soil under different treatments (*p* < 0.05), in the following order as CK < T_2_ < T_1_. The NH_4_^+^-N content in T_2_ was higher (1.83 and 1.12 times) than CK and T_1_, respectively. The NO_3_^−^-N content under CK was significantly higher (36.93 and 32.26%) than T_1_ and T_2_ treated plants. The significant differences in AP content in tobacco-growing soil under different treatments (*p* < 0.05) as CK < T_1_ < T_2_. The AP content of T_2_ was higher (1.80 and 1.24 times) than CK and T_1_, respectively (Figure 1). There was no significant difference in the contents of TN and AK among the treatments (*p* > 0.05).

### Diversity and richness analysis of soil bacterial communities

**Figure 2:**
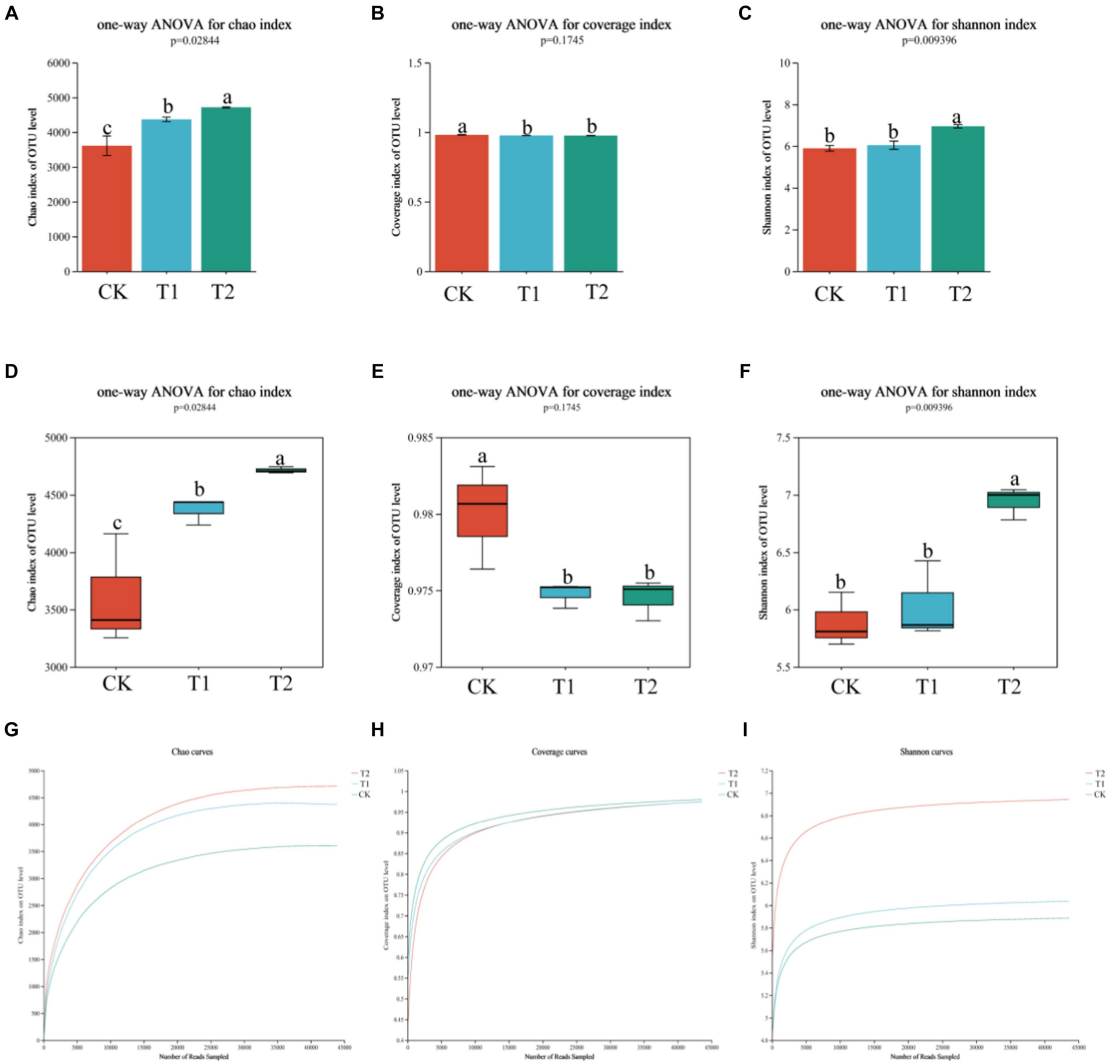
The analysis of bacterial α diversity in tobacco-planting soil with different previously cultivated crops. Different letters indicate significant differences between treatments (*p* < 0.05). **(A)** the bar graph of Chao1 index; **(B)** the bar graph of coverage index; **(C)** the bar graph of Shannon index; **(D)** the box graph of Chao1 index; **(E)** the bar graph of coverage index; **(F)** the bar graph of Shannon index; **(G)** the dilution curve of Chao1 index; **(H)** the dilution curve of coverage index; **(I)** the dilution curve of Shannon index.

A total of 1,097,678 high-quality sequences were obtained. It showed that in terms of soil bacterial community diversity and richness, T_1_ and T_2_ were similar, and no significant difference existed between treatments (Table 1). On the other hand, CK was significantly different compared to T_1_ and T_2_ treatments. The Chao1 index of the CK treatment was found 30.67 and 7.87% lower than T_1_ and T_2_ conditions. The Shannon index of CK was 18.02 and 15.09% lower than T_1_ and T_2_. Regarding the soil bacterial alpha diversity analysis, the similarity between T_1_ and T_2_ was higher, while the similarity between T_1_ and CK was lower as shown in Figure 2. The community coverage in CK was higher than T_1_ and T_2_, while CK community richness and diversity were found lower than T_1_ and T_2_ (Figure 2 and Table 1). At the same time, when the rarefaction curves were assessed, it tended to flatten towards the end, indicating that the sample sequencing data were high quality and could be analyzed in the next step.

**Table 1:** Tobacco-planting soil bacterial diversity indices.

Treatment	Chao1	Coverage	Shannon
CK	3607.37 ± 485.68^b^	0.98 ± 0.01^a^	5.88 ± 0.23^b^
T1	4370.13 ± 115.51^a^	0.97 ± 0.01^b^	6.03 ± 0.34^a^
T2	4713.89 ± 27.36^a^	0.97 ± 0.01^b^	6.94 ± 0.14^a^

Different letters indicate significant differences between treatments (*p* < 0.05).

**Figure 3:**
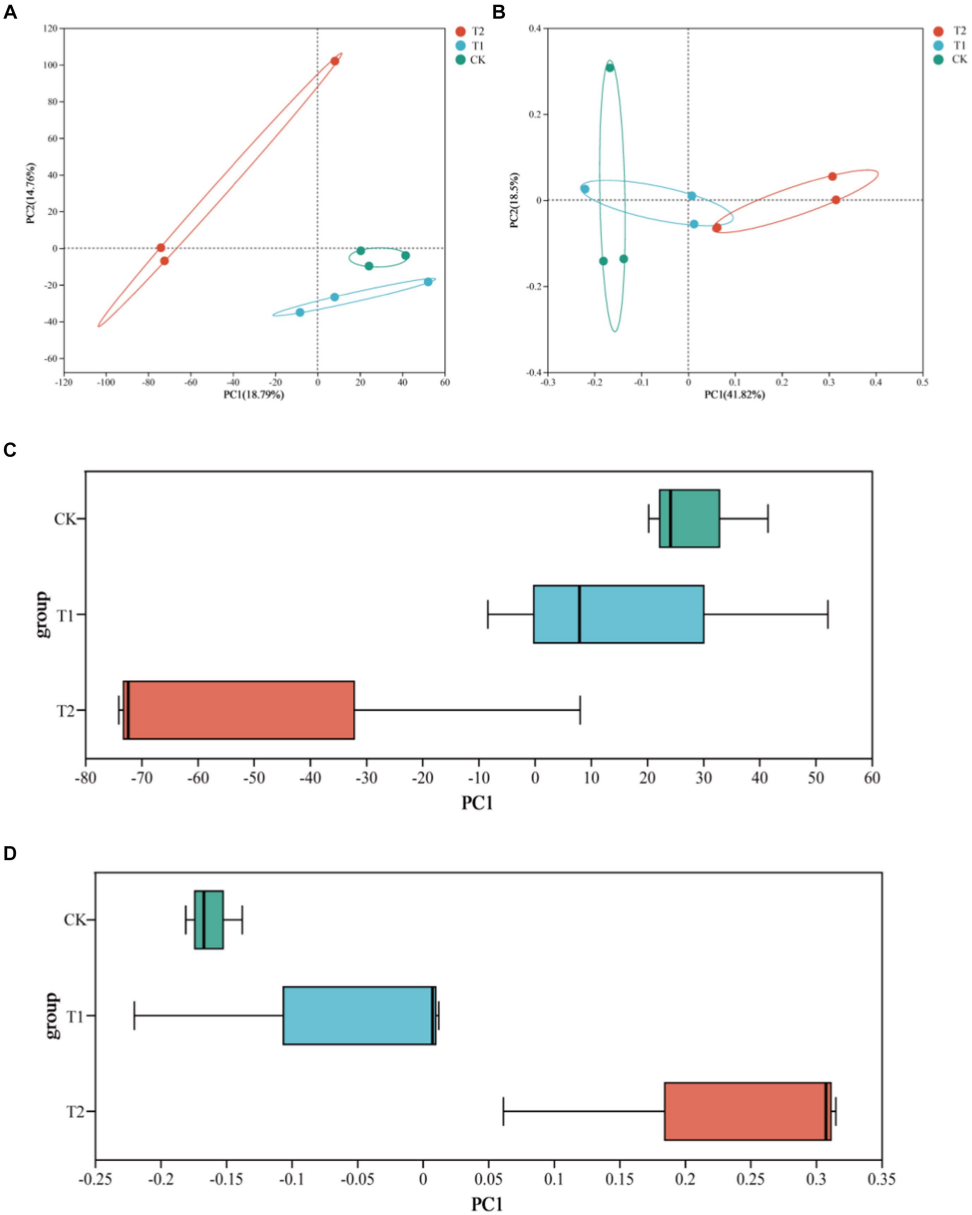
Analysis of bacterial β diversity in tobacco-planting soil with different cultivated crops. **(A)** the PCA of bacterial communities among the different treatments; **(B)** the PCoA of bacterial communities among the different treatments; **(C)** the PCA box graph of bacterial communities among the different treatments; **(D)** the PCoA box graph of bacterial communities among the different treatments.

Regarding the beta diversity analysis of soil bacterial community diversity and richness, PCA and PCoA revealed the similarities and differences of the bacterial communities among the different treatments. The box diagram more intuitively showed the interpretability value of the PC1 axis between the treatments and the dispersion of the distribution of the treatments on the PC1 axis. PCA and PCoA figures showed no crossover between CK and T_2_, indicating significant differences in soil bacterial community diversity and richness under both treatments (Figure 3).

### Analysis of soil bacteria species composition and their differences

**Figure 4:**
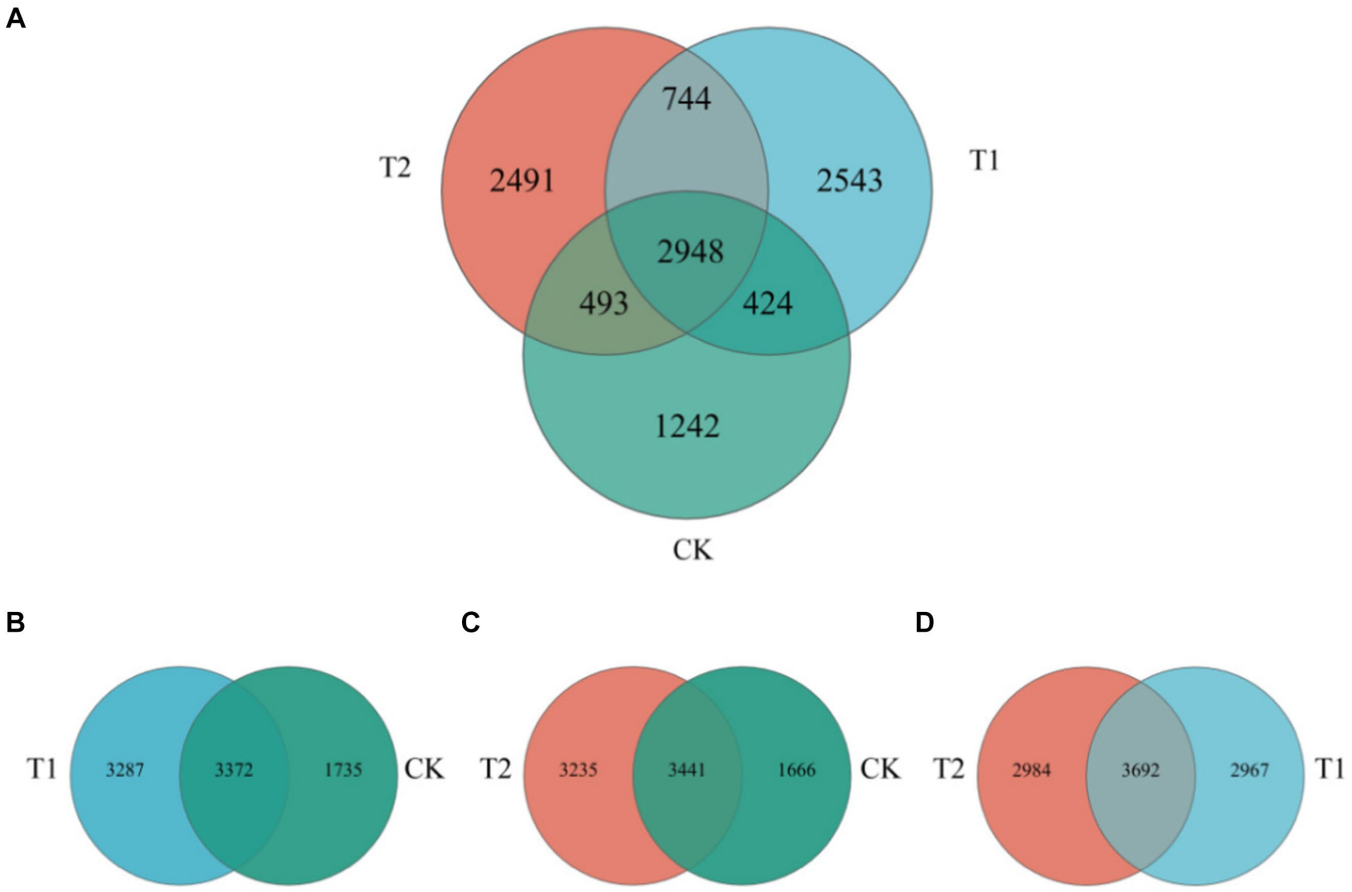
Venn diagram of bacterial species composition in tobacco-planting soil cultivated with different preceding crops. **(A)** the Venn of bacterial communities among CK, T1 and T2; **(B)** the Venn of bacterial communities among CK and T1; **(C)** the Venn of bacterial communities among CK and T2; **(D)** the Venn of bacterial communities among T1 and T2.

**Figure 5:**
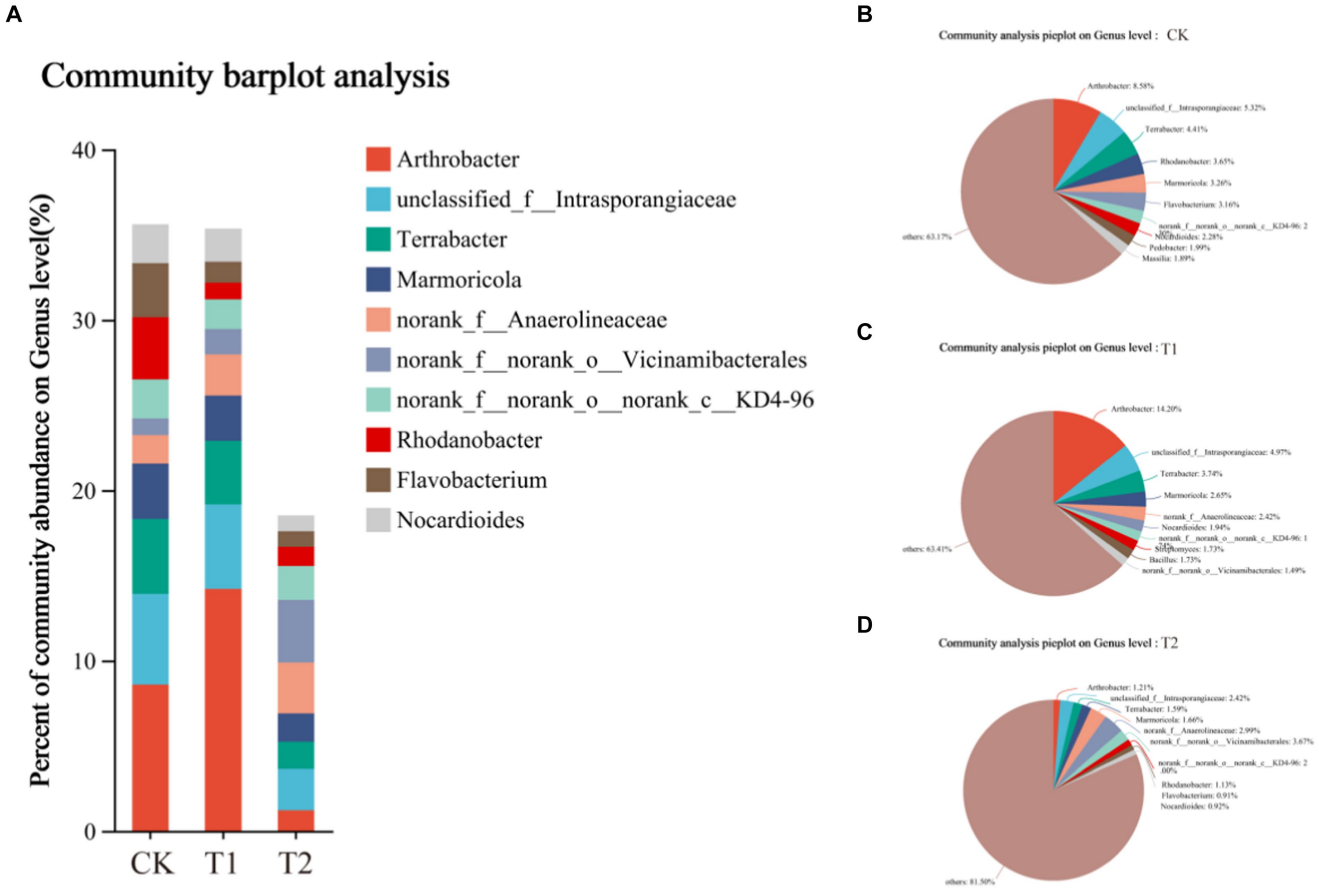
Composition of rhizosphere bacterial community in tobacco-planting soil cultivated with different preceding crops. **(A)** the community of barplot analysis; **(B)** the community analysis pieplot of CK bacterial communities on genus level; **(C)** the community analysis pieplot of T1 bacterial communities on genus level; **(D)** the community analysis pieplot of T2 bacterial communities on genus level.

The composition of the bacterial species of tobacco-planting soil previously cultivated with different crops. Through OTU analysis, 5,107 species were identified in CK, 6659 in T_1_, and 6,676 in T_2_ treatments. The species identified in CK were significantly lower than other treatments. Among the three treatments, 2,948 species were common, 1,242 were unique to CK, 2543 were unique to T_1_, and 2,491 were unique to T_2_ (Figures 4, 5). The comparison between CK and T_1_ revealed 3,372 common species, 1735 unique ones in CK, and 3,287 unique ones in T_1_. Comparing the both treatments (CK and T_2_), 3,441 common species were identified, 1,666 species unique to CK and 3,235 species unique to T_2_. Figure 5 shows the top ten most abundant bacterial genera in tobacco-planting soil under different preceding crops. The relative proportion of the ten most abundant dominant genera in tobacco-planting soil under different preceding crops was significantly different. The ten most dominant genera in abundance accounted for 36.83% of the total sequences in CK, 36.59% in T_1_, and only 18.50% in T_2_. *Arthrobacter* was the most differentially abundant strain among the three treatments. It accounted for 8.58% of all bacteria in CK, 14.20% in the T_1_ and 1.21% in T_2_ treatment, respectively.

**Figure 6:**
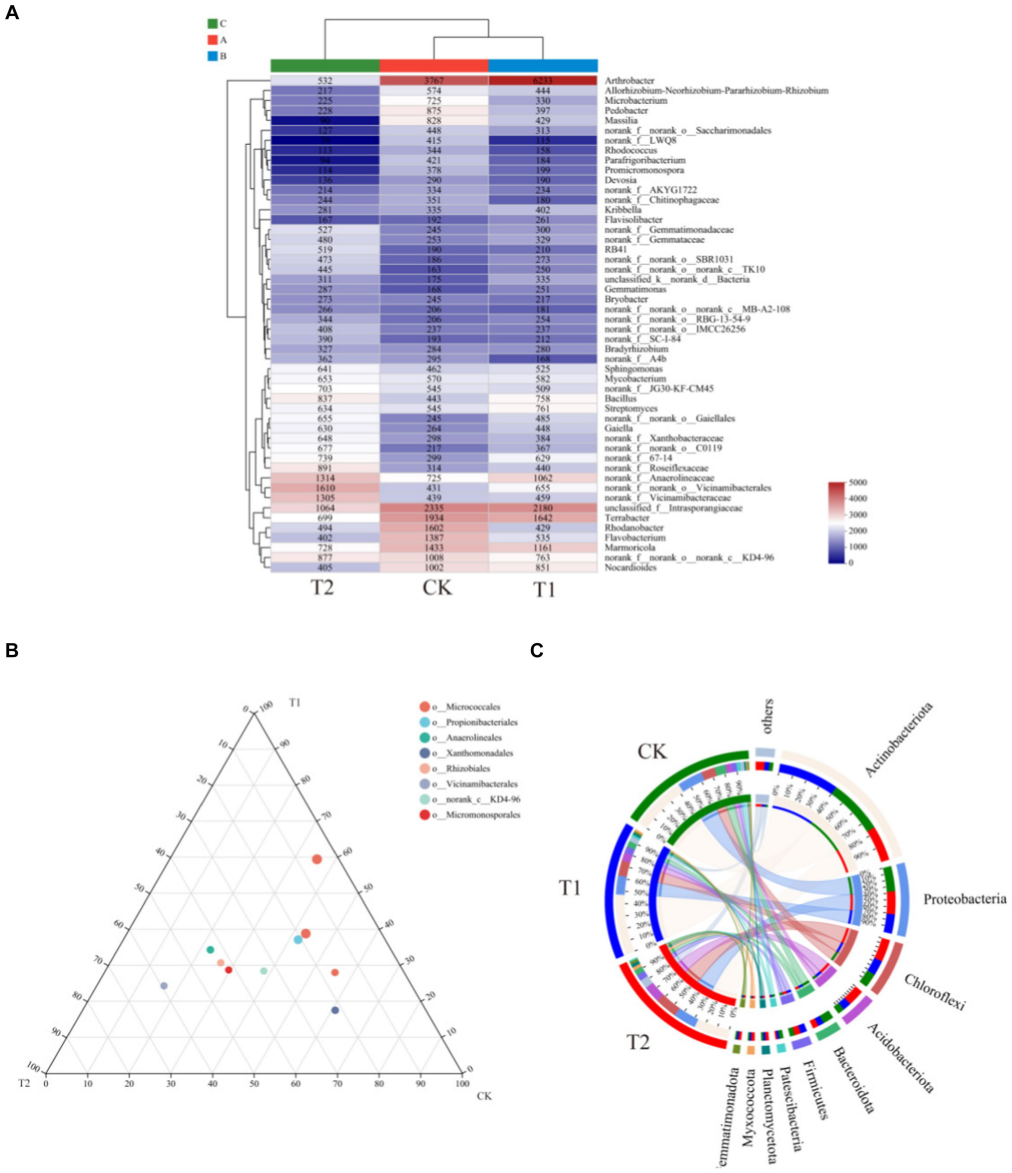
Relationship between rhizosphere bacterial community composition of the different preceding crops and tobacco-planting soil properties. **(A)** the heatmap of soil bacterial communities among different treatments; **(B)** the ternary analysis of CK, T1 and T2 on family level; **(C)** the circos analysis of CK, T1 and T2 on phylum level.

**Figure 7:**
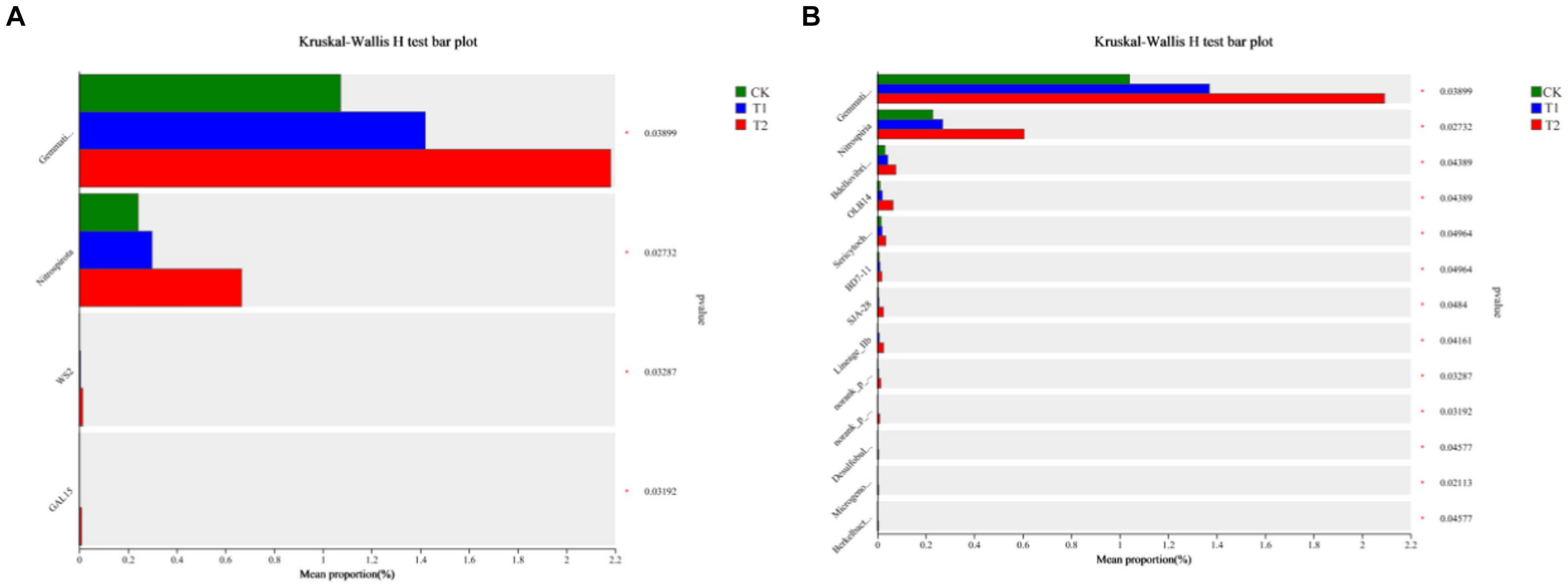
Difference analysis of bacterial species in tobacco-planting soil cultivated with different preceding crops. **(A)** the Kruskal-Wallis H test bar plot of CK, T1 and T2 on class level; **(B)** the Kruskal-Wallis H test bar plot of CK, T1 and T2 on phylum level.

**Figure 8:**
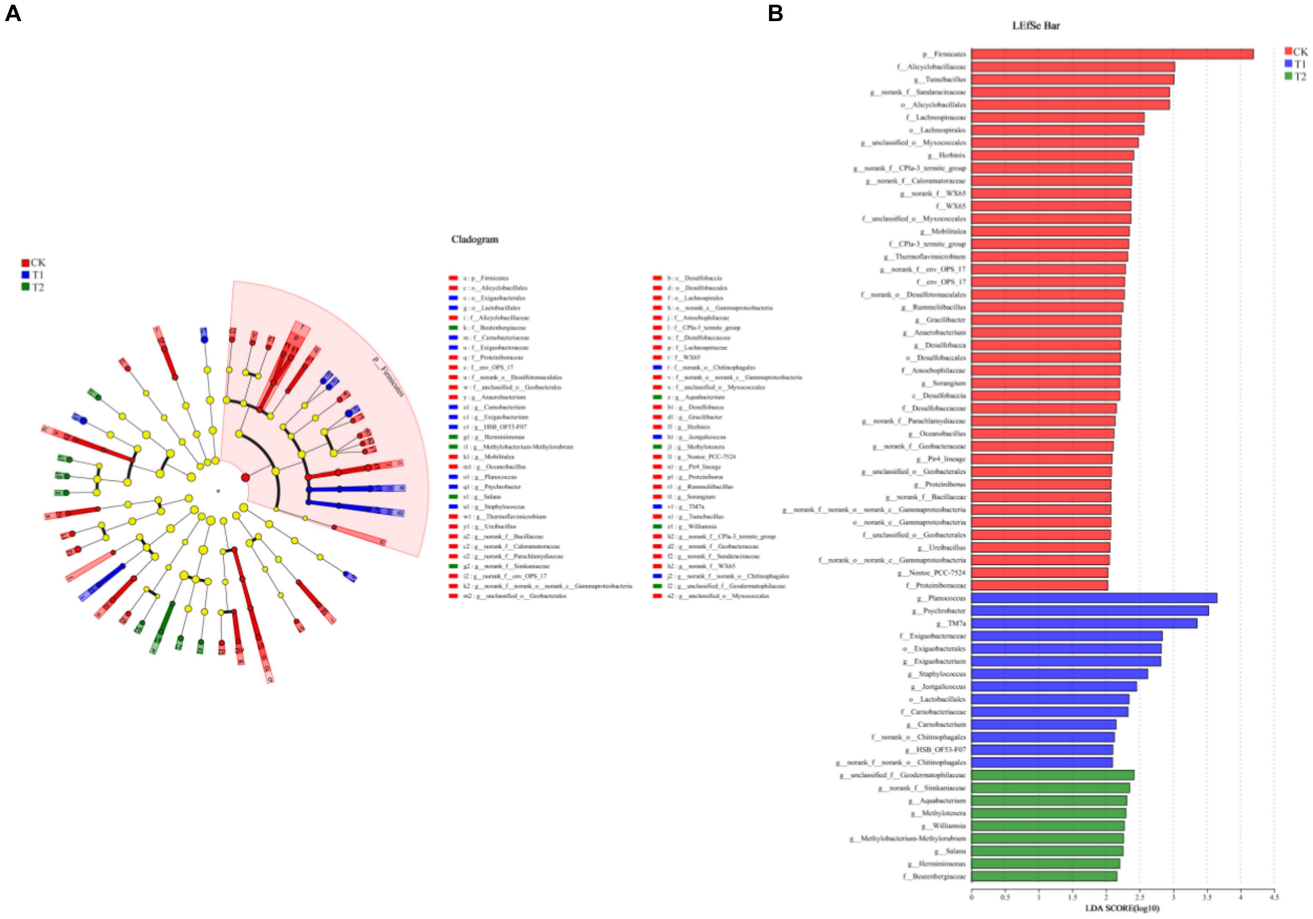
Species difference analysis of dominant bacterial species in tobacco-planting soil cultivated with different preceding crops. **(A)** the LEfSe multilevel species level tree from phylum to genus; **(B)** the LEfSE bar of LDA discriminant histogram.

Figure 6 indicates the species composition of the soil bacterial communities under different preceding crops, assessed with a heatmap, sample clustering tree analysis of treatment, species relationships, and a Ternary phase diagram. The species composition varied significantly among different treatments, but the species composition of bacteria under the CK and T_1_ treatments was generally similar (Figure 6). At the phylum level, the dominant bacteria under different treatments were identical, with *Actinobacteriota* accounting for 36.00% of all sequences in CK, 40.37% in T1, and 23.34% in T2. *Proteobacteria* was the second dominant bacterial phylum. Its relative abundance was found under the CK, T_1_, and T_2_ (38.17, 28.12 and 34.75%), respectively. The ternary phase diagram revealed the bacterial composition characteristics of 8 families of rhizosphere soil samples under control, barley and rapeseed. Circles of the same color in the figure represent the same family, and the circle area size represents their abundance. The results showed that the composition and distribution ratio of microorganisms in different samples differed, and the dominant strains under each treatment also differed. At the phylum level, *Gemmatimonadota, Nitrospirota*, WS2, and GAL15 were significantly different (*p* < 0.05) among the treatment, and the dominant phyla in T_2_ had a greater abundance than other treatments (Figure 7). The 13 most abundant classes reached a significant level at the class level. Through LEfSe difference analysis with an LDA threshold of 2, microorganisms with substantial differences were screened out in the three tobacco planting soils with different preceding crops (Figure 8).

### Correlation analysis of soil bacteria and the soil rhizospheric environment

**Figure 9:**
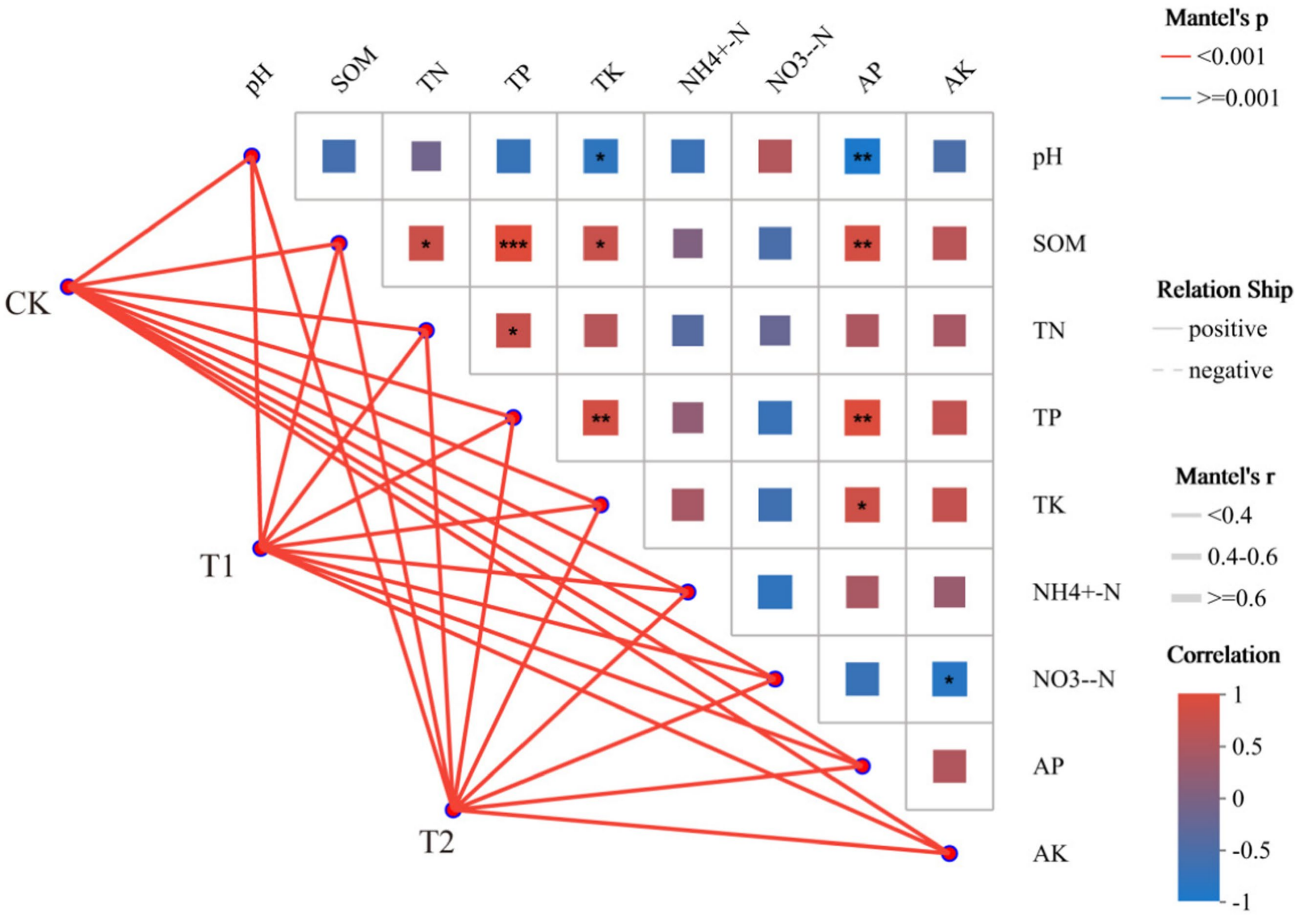
Correlation analysis between the different preceding crops and the tobacco-planting soil environment characteristics.

The correlation between soil environmental factors and bacterial community status in tobacco-planting soil cultivated with different preceding crops was assessed with a Mantel Test network heat map analysis (Figure 9). The lines in the figure represent the correlation between community and environmental factors and the heat map represents the correlation between environmental factors. The line thickness represents the magnitude of the correlation between the community and environmental factors, and positive and negative indicate the positive and negative correlations between the community and environmental factors. In the heat map, different colors represent positive and negative correlations. Colour depth represents the magnitude of positive and negative correlations, and asterisks in colour blocks represent significance. Soil environmental factors and bacterial community properties after cultivation with different preceding crops were positively correlated. At the same time, the correlations between environmental factors in tobacco-planting soil were different (Figure 9). SOM showed very significant positive correlation with TP (*p* < 0.001), while it showed no correlation with NH_4_^+^-N and NO_3_^−^-N. Among them, NO_3_^−^-N was not correlated with most environmental factors.

**Figure 10:**
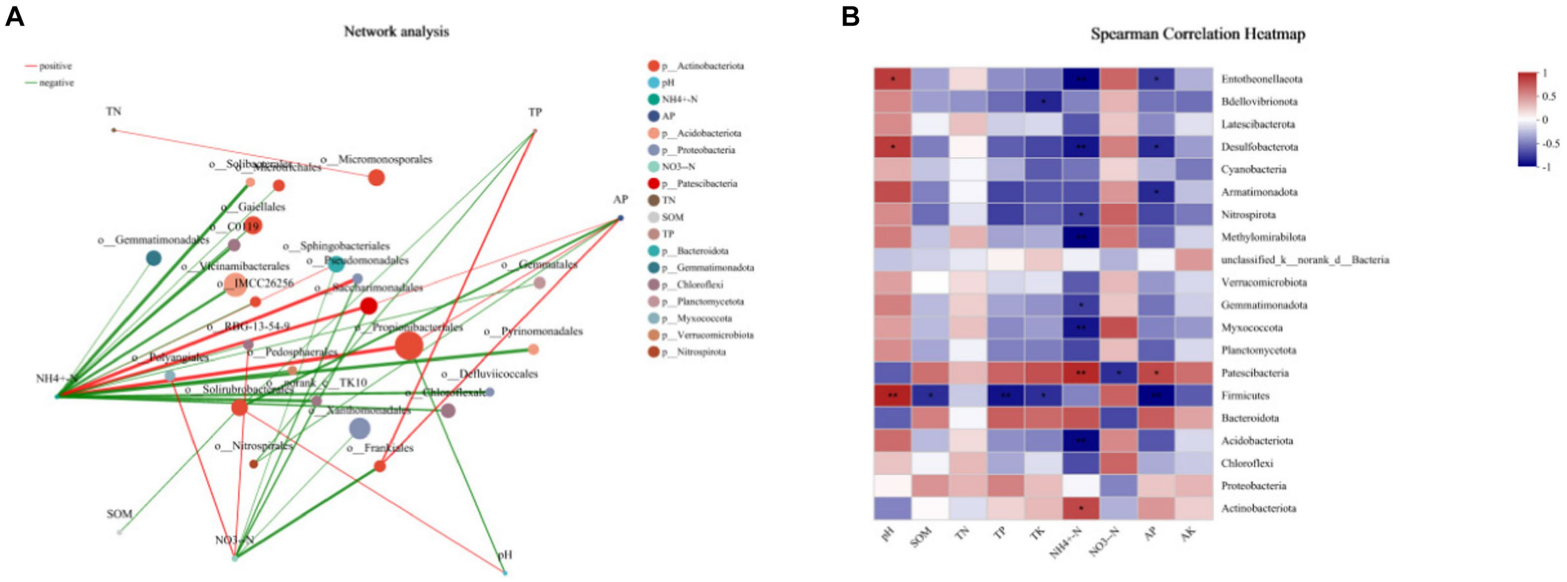
Correlation analysis between the tobacco soil environment characteristics and the dominant bacterial species. **(A)** the two-factor correlation network diagram between species and environmental factors; **(B)** the heatmap of correlations between species and environmental factors.

The two-factor correlation network diagram was used to analyze the correlation between bacterial species and environmental factors to facilitate the understanding of their interactions (Figure 10). The size of nodes represents the abundance of species, and different colors represent different species. The color of the line indicates a positive and negative correlation. Red indicates a positive correlation, and green indicates a negative correlation. The line’s thickness corresponds to the correlation coefficient’s magnitude, with thicker lines indicating stronger correlations between species. The more significant number of lines indicates a closer connection between the nodes. It could be seen from the figure that NH_4_^+^-N and NO_3_^−^-N were the environmental factors that exhibited a high correlation with bacteria in tobacco-growing soil. While *Saccharimonadales* was strongly correlated with NH_4_^+^-N and NO_3_^—^N. It showed a strong positive correlation with NH_4_^+^-N and a strong negative correlation with NO_3_^−^-N. *Propionibacteriales* and *Vicinamibacteriales* were the dominant bacterial phyla in the tobacco-planting soil and highly correlated with soil environmental factors.

## Discussion

Soil properties are affected by a variety of climatic factors, such as natural factors and artificial cultivation. Different regions and different planting patterns have a significant influence on the soil environment (Verma et al., 2023, 2024; Paes da Costa et al., 2024). As an important cash crop, tobacco cultivation requires better soil quality with rich macro and micro-nutrients. Therefore, suitable and sufficient soil nutrient levels could promote the growth of tobacco and improve its quality. Since tobacco is not a crop used in continuous cropping systems, farmers frequently employ crop rotation in the cultivation and production of tobacco. This study showed that selecting different preceding crops could lead to differences in the soil nutrient levels. Barley and rapeseed, as preceding crops, reduced the soil pH compared with fallow land. The soil pH suitable for growing flue-cured tobacco is weakly acidic, and acidic soil is conducive to tobacco growth (Zhang et al., 2016). Flue-cured tobacco has strong resistance to soil acidity and wide adaptability to various soil pH levels. The most suitable range of soil pH for producing high-quality tobacco leaves was 5.5–6.5 (Uwiringiyimana et al., 2023). Based on the results of this study, the pH of tobacco-planting soil after barley and rapeseed as preceding crops was nearly 6. It showed that barley and rapeseed could enhance the TN, TP, and TK contents in tobacco-planting soil but significantly reduce the NO_3_^−^-N content. The study of Lu et al. (2021) showed that the flue-cured tobacco crop thrives in nitrogen-rich conditions, and nitrate nitrogen was conducive to the growth and development of flue-cured tobacco in the early stage and the maturation and browning in the later stage. It showed that using barley and rapeseed as preceding crops would decrease NO_3_^−^-N content in tobacco-planting soil. Tobacco-planting soil will be more suitable for flue-cured tobacco growth, attention should be paid to NO_3_^−^-N fertilization during flue-cured tobacco planting.

The soil type and cultivar jointly influenced soil microbial community abundance and their metabolic potential in chickpea rhizosphere (Sneha et al., 2021). In this study, Illumina MiSeq high-throughput sequencing technology was used to assess the effects of different land use methods on soil bacterial community status. Significant differences were identified in bacterial diversity indices in tobacco-planting soil previously cultivated or not with different crops. Many studies have also found that the level of biodiversity was higher in farmland under crop rotation practices (Francioli et al., 2016; Bledsoe et al., 2020). This study showed that different preceding crops had different effects on the diversity of bacterial communities in soil. In terms of species richness, the Chao and Shannon indices in tobacco soil previously planted with rapeseed were higher than those previously planted with barley, and the community diversity was higher. It indicated that the tobacco-planting soil should be previously cultivated. It could promote the growth of certain bacterial species in the soil bacterial community, increasing the richness and abundance of soil bacteria. However, no previous planting inhibit the growth of some bacteria, resulting in a decline in soil bacterial richness. At the same time, selecting different crops as preceding crops would also affect soil bacterial communities differently. In this study, PCA, PCoA, and box diagram analysis of the bacterial communities in the tobacco planting soil under the three treatments revealed significant differences in the bacterial communities in the tobacco planting soil under the three treatments. The different land use and planting applications significantly impacted the soil microbial community properties (Reganold and Wachter, 2016).

Illumina’s high-throughput sequencing technology and bioinformatics analysis were used to compare the bacterial community abundance and genetic diversity in tobacco-planting soil cultivated with different preceding crops. The dominant bacteria in the three pre-treated tobacco-planting soils in this study included *Actinobacteriota, Proteobacteria*, and *Chloroflex*i, consistent with the similar findings (Wang et al., 2021). It indicates the composition of soil bacterial communities at the phylum level was similar under different land use levels. Still, their relative abundance was different, which might be due to differences in vegetation types and soil nutrients’ forms and contents (Hu et al., 2021; Xie et al., 2021; Bai et al., 2022). It showed that the bacterial species in tobacco-planting soil were more abundant after cultivation with preceding crops.

Moreover, the dominant bacterial species in the tobacco-planting soil, when rapeseed was the preceding crop, accounted for the lowest proportion of all sequences, indicating a more uniform distribution of bacterial species during this situation. *Actinobacteriota* mostly saprophytic bacteria and can secrete large amounts of extracellular hydrolases, degrade insoluble organic matter in the soil for cell metabolism, and play an essential role in soil material cycling and improvement (Bhatta Kaudal and Weatherley, 2018). *Actinobacteriota* were widely distributed in terrestrial ecosystems, especially in arid soils (Naz et al., 2023; Ngamcharungchit et al., 2023). Present result showed that *Arthrobacter* was the dominant bacteria in the tobacco soil. Barley plants planted as a preceding crop, and their abundance was similar to that of barley not planted as a preceding crop.

The soil microbial community properties and diversity might be strongly influenced by soil physical and chemical properties, such as soil moisture and soil nutrient availability, and could reflect changes in soil ecological processes (Habteselassie et al., 2022; Li et al., 2022). This study showed that preceding crop cultivation could significantly affect the physicochemical properties of tobacco-plant soil, overall depends on the crop types. The cropping practices changed the chemical properties of the rhizosphere soil and the composition, status, and diversity of the rhizosphere microbial communities (Liu et al., 2022). Meanwhile, the two-factor correlation network results showed that NH_4_^+^-N and NO_3_^−^-N were the environmental factors with a high correlation with bacteria in the tobacco-planting soil. While *Saccharimonadales* was strongly correlated with NH_4_^+^-N and NO_3_^−^-N, it presented strong positive correlation with NH_4_^+^-N and a strong negative correlation with NO_3_^−^-N. *Propionibacteriales* and *Vicinamibacteriales* were the dominant phyla in tobacco-planting oil and highly correlated with soil environmental factors. Some studies indicated that soil bacteria widely involved in the soil nitrogen cycle. The changes in different types during the growth and decay of previous crops would cause changes in soil nitrogen, further affecting the *proteobacteria* group closely related to nitrogen fixation (Zhao et al., 2021; Yang et al., 2023). It showed that NH_4_^+^-N and NO_3_^−^-N were the two environmental factors most strongly correlated with bacteria in tobacco-planting soil, and the changes of NH_4_^+^-N and NO_3_^−^-N significantly change the diversity and abundance of bacteria. At the same time, *Firmicutes* exhibited a strong correlation with environmental factors of tobacco planting soil. Therefore, it is more sensitive to changes in the soil environment.

## Conclusion

This study investigated the nutrient status, bacterial community composition, and properties of tobacco-planting soil previously cultivated with barley rapeseed or not cultivated before tobacco planting. It showed that different preceding crops significantly changed tobacco-planting soil nutrient status, physical and chemical properties. The different preceding crops had different effects on soil nitrogen status, bringing to our attention the importance of choosing and applying various forms of nitrogen fertilizer during the cultivation of crops preceding tobacco planting. The significant differences in the diversity and richness of bacterial communities in tobacco-planting soil under different previous crops, and some strains with high correlation with soil environmental factors were selected. It showed that the rapeseed plants are the better option to preceding tobacco crop cultivation and management.

## Data availability statement

The datasets presented in this study can be found in online repositories. The names of the repository/repositories and accession number(s) can be found at: https://www.ncbi.nlm.nih.gov/, SUB14128373.

## Author contributions

ML: Data curation, Methodology, Supervision, Writing – original draft, Writing – review & editing. RX: Conceptualization, Investigation, Software, Writing – original draft. DW: Resources, Visualization, Writing – original draft. YH: Formal analysis, Project administration, Validation, Writing – original draft. KG: Writing – original draft. LY: Writing – original draft. JZ: Conceptualization, Data curation, Writing – original draft. SG: Conceptualization, Writing – original draft. JS: Funding acquisition, Resources, Visualization, Writing – review & editing. YJ: Project administration, Supervision, Validation, Visualization, Writing – review & editing.

## Footnotes

^1^ https://cloud.majorbio.com/page/tools/
